# Characterization of a sandwich ELISA for quantification of total human soluble neuropilin‐1

**DOI:** 10.1002/jcla.22944

**Published:** 2019-06-20

**Authors:** Elisabeth Gadermaier, Manfred Tesarz, Jacqueline Wallwitz, Gabriela Berg, Gottfried Himmler

**Affiliations:** ^1^ The Antibody Lab GmbH Vienna Austria; ^2^ Biomedica Medizinprodukte GmbH Vienna Austria

**Keywords:** biomarker, ELISA, sandwich ELISA, soluble human neuropilin‐1, soluble neuropilin‐1

## Abstract

**Background:**

Neuropilin‐1 (NRP1) is a highly interactive molecule that exists as transmembrane and soluble isoforms. Measurement of circulating levels of soluble NRP1 (sNRP1) in human serum and plasma has proven to be difficult due to present matrix interferences and due to the lack of a reliable technique.

**Methods:**

We developed a highly specific and sensitive sandwich ELISA assay for total sNRP1 quantification in peripheral blood, and we validated the test according to ICH guidelines. The linear epitopes of the employed polyclonal and monoclonal anti‐human NRP1 antibodies were mapped with microarray technology. We included a sample pre‐treatment step with guanidine hydrochloride (GuHCl) to release sNRP1 from existing interferants.

**Results:**

The ELISA assay which is calibrated with sNRP1 isoform 2 and covers a calibration range from 0.375 to 12 nmol/L detects sNRP1 in human serum and plasma (heparin, EDTA, and citrate). Multiple linear epitopes recognized by the polyclonal coating antibody are distributed over the whole sNRP1 sequence. The monoclonal detection antibody binds to a linear epitope which is in the N‐terminal region of the a1 domain of human sNRP1. Assay parameters like precision (intra‐assay: 6%), dilution linearity (95%‐115%), specificity (98%), and spike recovery (81%‐109%) meet the international standards of acceptance.

**Conclusion:**

Our novel sandwich ELISA provides a reliable tool for the quantitative determination of total human sNRP1. The assay detects free and previous ligand‐bound total NRP1.

**Abbreviations**
GuHCl: guanidine hydrochloride
NRP1: neuropilin‐1
sNRP1: soluble neuropilin‐1

## 1 INTRODUCTION

Neuropilin‐1 (NRP1) is a single‐pass transmembrane glycoprotein of 923 amino acids first described in 1987 by Fujisawa et al.^1^ It exists as transmembrane isoform 1, and as soluble NRP1 (sNRP1) isoforms 2 and 3 generated by alternative splicing. Isoform 1 is composed of a large extracellular region, a short transmembrane domain, and a short cytoplasmic tail without enzymatic activity. The extracellular region consists of five subdomains, that is, a1 and a2 (CUB motifs), b1 and b2 (coagulation factor V/VIII domains), and c (MAM domain). While the MAM domain is suggested to assist in receptor assembly and regulation of signaling,^2^ the a and b domains are responsible for binding of ligands. The pivotal role of NRP1 in key physiological processes is illustrated by the nature of its ligands. These include chemorepellent proteins for axons like semaphorin 3A (SEMA3A)^3^ as well as regulators of vasculogenesis, angiogenesis, and vascular remodeling, like members of the VEGF ligand family, for example, placental‐like growth factor (PLGF) and the VEGF_165_ isoform of vascular endothelial growth factor A.^4^ However, despite its important role in development, NRP1 also plays an important role in pathological conditions like cancer^5^, ^6^ or nephropathies.^7^

Alternative splicing or ectodomain shedding by the metalloprotease ADAM10 leads to the release of the extracellular a and b regions as soluble NRP1 (sNRP1).^8^, ^9^, ^10^, ^11^ The expression of transmembrane or soluble NRP1 is thereby cell‐dependent,^8^ and the activities of both forms are opposing. Transmembrane NRP1 functions as co‐receptor that mediates signaling in a variety of cell types by binding to proteins containing a PDZ domain.^3^, ^4^, ^12^ In contrast, sNRP1 binds to the same ligands as transmembrane NRP1, but acts as decoy. In this context, sNRP1 was described as VEGF_165 _antagonist that has antitumor activities.^8^

To study the function of NRP1 and its role as a potential biomarker in various diseases, we developed a sandwich ELISA for robust and reliable quantification in human blood. We included a sample pre‐treatment step to overcome interferences, which finally allowed us to measure free and previous ligand‐bound total NRP1. We unveiled linear epitopes of the employed monoclonal and polyclonal anti‐human NRP1 antibodies to provide best possible insight into the binding properties of the antibodies utilized in the assay. Furthermore, we validated the assay based on the principles of bioanalytical validation defined by the ICH harmonized tripartite guideline Q2 (R1).

## 2 MATERIALS AND METHODS

### 2.1 Serum and plasma samples

Venous blood samples were obtained from apparently healthy subjects after informed consent was given. Blood collection tubes for serum or plasma (citrate, heparin, and EDTA) were used for sample collection. Samples were incubated 10 minutes at room temperature before centrifugation (10 minutes, 2000 g). Samples were stored at −20°C. Testing was done in an anonymized manner.

### 2.2 Effect of GuHCl on high‐affinity bound ligands to sNRP1

Measurements were performed on an Octet K2 instrument (ForteBio, Pall life sciences) at 25°C. For amine coupling, two AR2G sensors (ForteBio) were activated for 10 minutes with a mixture of 1‐ethyl‐3‐(3‐dimethylaminopropyl carbodiimide) hydrochloride and N‐hydroxysulfosuccinimide and further loaded for 15 minutes with monomeric sNRP1 diluted in sodium acetate (pH 4). Then, sensors were deactivated for 10 minutes with 1 mol/L ethanolamine‐HCl (pH 8.5). Sensor 1 was incubated with recombinant human VEGF_165_ (Enzo Life Sciences; 5 µg/mL in PBS) for 10 minutes, followed by a dissociation period of 3 minutes in PBS. Reference sensor 2 was incubated in parallel with PBS. Regeneration of both sensors was performed with 3 mol/L GuHCl in PBS for 5 minutes, followed by a stabilization in PBS for 2 minutes.

### 2.3 Antibodies for sandwich ELISA development

The polyclonal sheep anti‐human NRP1 antibody was raised against and affinity‐purified with full‐length NRP1. The monoclonal mouse anti‐human NRP1 was also raised against full‐length NRP1, and purification was done via caprylic acid/ammonium sulfate precipitation.

### 2.4 Setup of the human sNRP1 sandwich ELISA

Plates were coated with 150 µL polyclonal sheep anti‐human NRP1 antibody overnight at 4°C. After aspiration, wells were blocked with blocking solution for 3 hours at room temperature. Then, wells were aspirated and dried. For calibration of the assay, sNRP1 isoform 2 (0.375‐12 nmol/L), expressed in a mouse myeloma cell line, was spiked into sNRP1‐free human serum matrix, that was generated by immunoaffinity chromatography depletion with a polyclonal anti‐human NRP1 antibody. Samples, control sera, and calibrator (10 µL) were pre‐treated with the chaotrope reagent guanidine hydrochloride (GuHCl). Briefly, samples were pre‐diluted 1:2 with GuHCl with a final concentration of 3 mol/L and incubated for 30 minutes at room temperature followed by 1:10 dilution with protein‐based assay buffer. Wells were pre‐filled with 50 µL protein‐buffered assay buffer. Thereafter, 50 µL of the pre‐treated samples, controls, and calibrator were pipetted together with 50 µL of the biotinylated mouse monoclonal anti‐human NRP1 detection antibody to the coated and blocked wells. After a two hours incubation, wells were washed for five times with wash buffer and 150 µL of the conjugate solution consisting of horseradish peroxidase‐labeled streptavidin was added for 1 hour to allow complex formation with the biotinylated detection antibody. After subsequent washing, 150 µL of the substrate solution was added. The enzymatic reaction catalyzing the color change of the substrate was stopped after 30 minutes incubation in the dark by adding 50 µL stop solution. The absorbance was measured with a microplate reader (BioTec) at 450 nm with an absorbance correction at 630 nm. A dose‐response curve of the absorbance vs the concentration of the calibrator was generated with a 4PL algorithm. The concentration of samples was calculated from the dose‐response curve. Our assay has been commercialized by Biomedica (Bi‐20409).

### 2.5 Influence of GuHCl sample pre‐treatment on measured sNRP1 concentrations

The influence of varying concentrations of GuHCl on sNRP1 quantification in samples was tested by pre‐treating human sera from different human donors with a final concentration of 3 mol/L, 1 mol/L, 0.15 mol/L, 0.05 mol/l, and 0 mol/L GuHCl. Further, eight serum samples were measured without GuHCl pre‐treatment and with varying incubation times of the detection antibody. Another three sera were pre‐treated with a final concentration of 3 mol/L GuHCl before measurement with varying detection antibody incubation times. Differences of NRP1 concentrations were analyzed with Wilcoxon rank testing.

### 2.6 Determination of assay characteristics

Validation was performed according to ICH guidelines. For the calculation of the lower limit of quantification (LLOQ), standard 2 (0.375 nmol/L) was diluted in twofold steps in protein‐based assay buffer and measured in five replicas per dilution. To calculate intra‐assay precision, seven serum samples were measured in replicates of five by one operator within one kit lot. For inter‐assay precision, seven serum samples in nine replicates within three kit lots were measured by three operators. To analyze assay specificity, samples from four different matrices (serum, EDTA plasma, heparin plasma, and citrate plasma) were tested with and without prior addition of a molar surplus of coating antibody acting as competitor. The accuracy of the assay was determined by spiking 6 nmol/L recombinant sNRP1 into serum, EDTA plasma, heparin plasma, and citrate plasma. Dilution linearity was assessed by diluting serum, EDTA plasma, heparin plasma and citrate plasma 1 + 1, 1 + 3 and 1 + 7 in protein‐based assay buffer. Stability of endogenous sNRP1 was determined by storing samples at different conditions (overnight on 4°C, one or three hours on room temperature). Further, freeze‐thaw stress was applied on endogenous and recombinant sNRP1 for one to five freeze‐thaw cycles.

### 2.7 Linear epitope mapping of both antibodies employed in the human sNRP1 assay

The sequence of human sNRP1 was covered by 623 synthetic linear 15mer peptides with an overlap of 14 amino acids. Peptides were printed on a microarray in duplicates (Pepperprint GmbH). After blocking, the array was incubated overnight with 1 µg/mL of coating or detection antibody. Bound antibodies were traced with donkey anti‐sheep IgG or with goat anti‐mouse IgG, respectively, both labeled with DyLight680. Arrays were scanned on a LI‐COR Odyssey Imaging System. Quantification of spot intensities was performed with PepSlide^®^ Analyzer Software based on 16‐bit tiff files. Median intensities of duplicates were averaged, and intensities with variations >40% were zeroed.

### 2.8 Surface representation of sNRP1

A three‐dimensional model of mouse sNRP1 obtained by X‐ray diffraction (PDB ID: 4GZ9) was used to visualize the domains a1a2b1b2, the linear epitopes identified by microarray analysis, and the binding sites of three NRP1‐binding molecules (SEMA3A,^3^, ^13^ VEGF‐A,^14^, ^15^ as well as of heparin 15) with the graphical analysis tool Cn3D.^16^

### 2.9 Testing of interferences on sNRP1 detection

Recombinant human sNRP1 (1.5 nmol/L) spiked in sNRP1‐depleted serum matrix was tested in the novel sNRP1 ELISA with and without GuHCl sample pre‐treatment. The sample was further tested with and without prior addition of a molar surplus of the NRP1 ligands SEMA3A (R&D Systems) or VEGF‐A_165 _(Enzo Life Sciences).

Interferences of SEMA3A or VEGF‐A_165_ with sNRP1 binding of the monoclonal anti‐human NRP1 detection antibody employed in the sandwich ELISA were further tested in bio‐layer interferometry measurements. First, dissociation rates of these two NRP1 ligands were tested. Therefore, biotinylated sNRP1 (4 µg/mL) diluted in PBS was loaded to two streptavidin sensors (ForteBio). Sensors were then either incubated for 10 minutes with 10 µg/mL VEGF‐A_165_ or SEMA3A. Dissociation was performed in PBS for 15 minutes. To further investigate if SEMA3A or VEGF‐A_165_ binding to sNRP1 interferes with antibody binding, four additional sensors were loaded with sNRP1 as described. Two sensors were then either incubated with 10 µg/mL VEGF‐A_165_ or SEMA3A for 10 minutes, while two other sensors were incubated with PBS alone acting as reference. After a one‐minute stabilization phase, association of the monoclonal antibody (2 µg/mL in PBS) was performed for 10 minutes for all four sensors, followed by a 15 minutes dissociation phase.

### 2.10 Statistics

Statistical analyses were carried out with GraphPad Prism 6 software. Values of *P* < 0.05 were considered as statistically significant.

## 3 RESULTS

### 3.1 GuHCl pre‐treatment of samples removes interferants and stabilizes sNRP1 measurement

**Figure 1:**
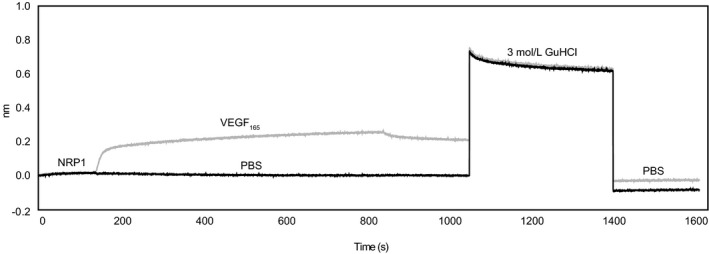
The effect of GuHCl on ligand‐bound sNRP1 was tested with bio‐layer interferometry. sNRP1 was immobilized to two AR2G sensors. Sensor 1 (gray) was incubated with the sNRP1‐ligand VEGF‐A_165_, sensor 2 (black) with PBS. Regeneration was performed with 3 mol/L GuHCl, followed by stabilization in PBS. Signal intensities are shown on the *y*‐axes (nm) vs the time (s, *x*‐axes)

Neuropilin‐1 is strongly interacting with other molecules. Therefore, the influence of the strong chaotrope GuHCl on ligand binding to NRP1 was tested in bio‐layer interferometry measurement with the high‐affinity NRP1 ligand VEGF‐A_165_. VEGF‐A_165 _stably bound to sensor‐immobilized sNRP1 but was completely removed after regeneration with a high concentration (3 mol/L) of the strong chaotrope GuHCl (Figure 1).

**Figure 2:**
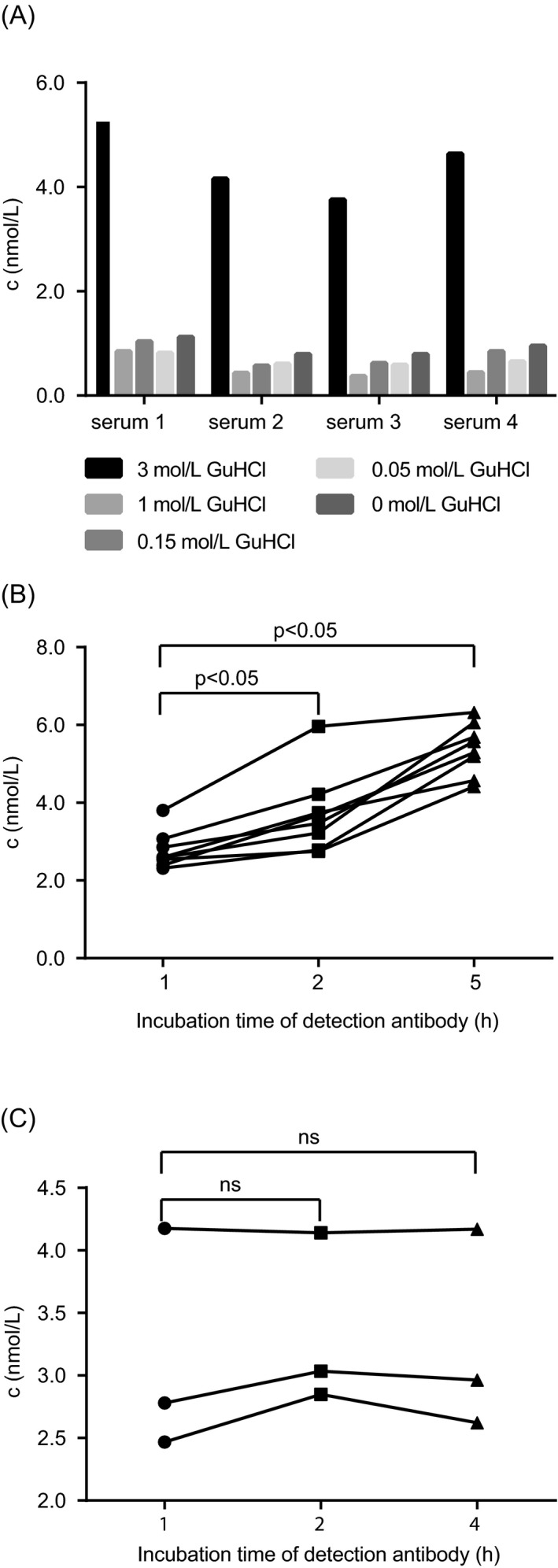
A, Four sera (*x*‐axis) were tested with 1:2 pre‐dilution with assay buffer containing varying concentrations of GuHCl (3 mol/L‐0 mol/L). B, Eight sera diluted in assay buffer without GuHCl were tested with different incubation times of the detection antibody (*x*‐axis, 1‐5 h). C, Three sera diluted in assay buffer containing 3 mol/L GuHCl were tested with different detection antibody incubation times (*x*‐axis, 1‐4 h). sNRP1 concentrations (nmol/L) are shown on the *y*‐axes. Significant deviations (*P* < 0.05) of concentrations are indicated (ns: not significant)

The relevance of this result in an immunoassay setting was analyzed during ELISA development. Serum pre‐treatment with increasing concentrations (0‐3 mol/L) of GuHCl prior to testing influenced sNRP1 levels. In detail, when 3 mol/L GuHCl was used for pre‐treatment, measured sNRP1 concentrations were 6.3‐fold higher compared to sNRP1 concentrations in samples that were untreated (0 mol/L) or treated with 0.05‐1 mol/L GuHCl (Figure 2A). Furthermore, measurement of non‐treated samples with longer incubation times of the detection antibody (1, 2, 5 hour) led to a significant increase of sNRP1 concentration over time due to non‐parallel OD increases of standard and samples (median serum sNRP1: 1 hour 2.6 nmol/L, 2 hour 3.6 nmol/L, 5 hour 5.4 nmol/L), an effect that was not observed with longer incubation times of coating antibody and samples (data not shown; Figure 2B). However, pre‐treatment of samples with 3 mol/L GuHCl stabilized sNRP1 concentrations during increasing detection antibody incubation times (median serum sNRP1: 1 hour 2.8 nmol/L, 2 hours 3.0 nmol/L, and 4 hours 3.0 nmol/L; Figure 2C).

### 3.2 Key parameters of the human sNRP1 sandwich ELISA

**Figure 3:**
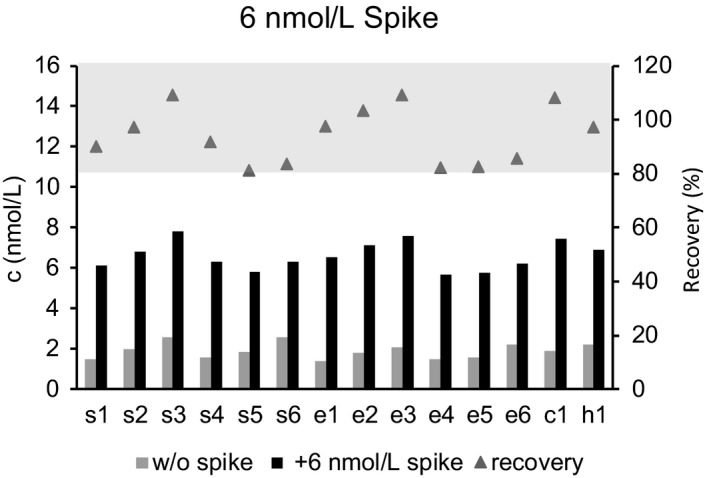
Six serum, six EDTA, one citrate, and one heparin plasma sample were tested without (gray bar), and with (black bar) a 6 nmol/L spike with recombinant sNRP1. Concentrations are shown (left *y*‐axis, nmol/L). Percent recoveries were calculated and are shown for each sample as triangles (right *y*‐axis). The gray area on top of the chart indicates the accepted recovery range between 80% and 120%

**Figure 4:**
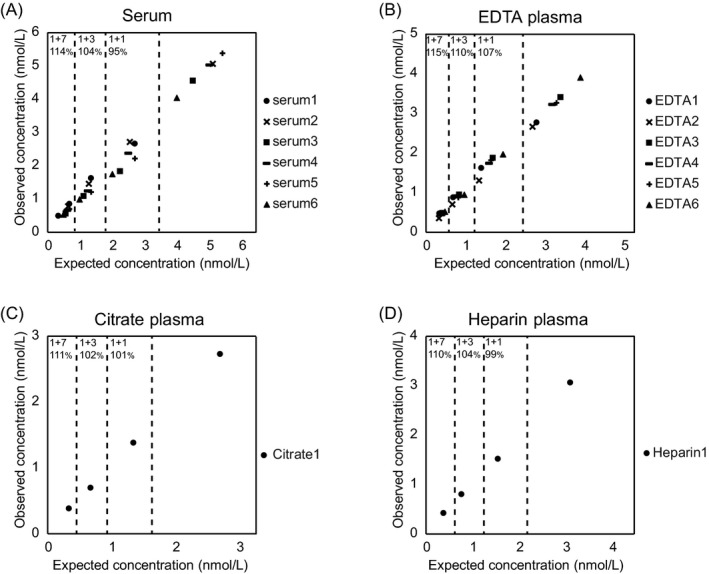
Six serum (A), six EDTA (B), one citrate (C), and one heparin (D) plasma sample were tested without dilution, and in 1 + 1, 1 + 3, and 1 + 7 dilution steps. Expected concentrations (*x*‐axes) were plotted vs observed concentrations (*y*‐axes). Mean percent recoveries compared to undiluted samples are shown on top of each chart

**Figure 5:**
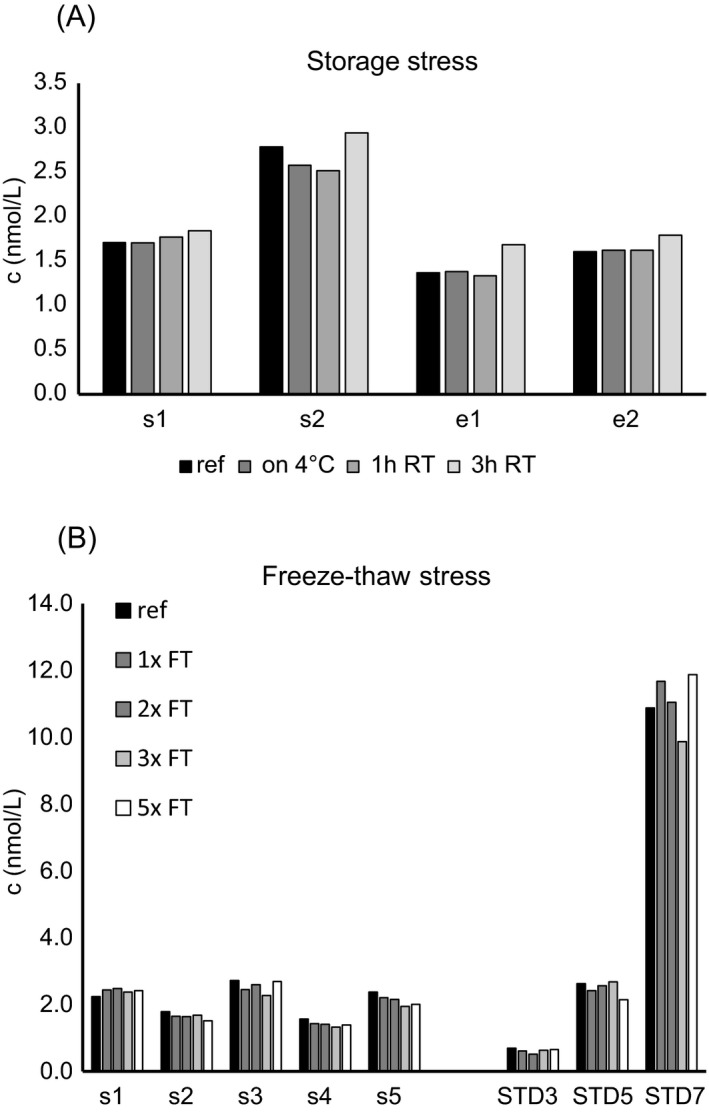
Stability of endogenous and recombinant sNRP1. A, Two serum (s) and two EDTA (e) plasma samples were stored over night at 4°C, or one or three hours at room temperature prior to assaying. B, Fife sera (s) and three calibrators (standard, STD) were subjected to zero‐five freeze‐thaw cycles prior to assaying

The human sNRP1 sandwich ELISA uses a polyclonal sheep anti‐human NRP1 antibody for capturing sNRP1 and a labeled monoclonal mouse anti‐human NRP1 antibody for detection. The calibrator sNRP1 isoform 2 defines the dynamic range of the assay between 0.375 nmol/L and 12 nmol/L. The quantification limit (LLOQ), as the lowest concentration with a percent CV of <25% and a percent backfit (ie, the ratio of the mean recalculated concentration to the theoretical concentration) between 75% and 125%, was defined as 0.09 nmol/L. The CV of intra‐assay precision ranged from 3%‐10% (mean: 6%) and from 8%‐15% (mean: 10%) for inter‐assay precision (Table 1). To show that the assay is specific for sNRP1, and to exclude interferences with unspecific matrix components, competition experiments with the anti‐NRP1 coating antibody were performed. In case of insufficient specificity, the competitor antibody, although applied in molar surplus, would bind to matrix components, and signal reduction would be impossible. Our results indicate that all samples could be competed with a mean competition of 98%, ranging from 84% to 100%, indicating that the assay is specific only for sNRP1 (Table 2). The accuracy of the assay was evaluated by adding a high concentration of recombinant sNRP1 (6 nmol/L) to different human serum and plasma samples. Calculated recovery ranged from 81% to 109% indicating high assay accuracy (Figure 3). Further, different serum and plasma samples were diluted 1 + 1, 1 + 3, and 1 + 7. The mean recovery for all dilution steps in the measured matrices ranged from 95% to 115% showing linear behavior across the dynamic assay range (Figure 4). To determine stability of endogenous sNRP1, different storage conditions (overnight 4°C, one/three hours room temperature) were applied to four samples (two sera, two EDTA plasma) prior to assaying. Endogenous sNRP1 was stable when incubated overnight at 4°C or one hour at room temperature, but longer incubation at room temperature should be avoided (Figure 5A). Testing of freeze‐thaw stability of endogenous (five sera) and recombinant sNRP1 revealed that samples and calibrator are stable for at least five times freeze‐thaw (Figure 5B).

**Table 1:** Determination of intra‐assay and inter‐assay precision

	Mean (nmol/L)	SD (nmol/L)	Precision (% CV)
Intra‐assay (n = 5)			
Serum 1	2.6	0.22	8
Serum 2	2.9	0.29	10
Serum 3	2.4	0.09	4
Serum 4	1.6	0.06	4
Serum 5	2.1	0.07	3
Serum 6	1.2	0.05	4
Serum 7	3.2	0.26	8
		Mean % CV	6
Inter‐assay (n = 9)			
Serum 1	2.6	0.22	9
Serum 2	2.8	0.41	15
Serum 3	2.4	0.24	10
Serum 4	1.7	0.17	10
Serum 5	2.2	0.18	8
Serum 6	1.1	0.13	11
Serum 7	3.3	0.33	10
		Mean % CV	10

Note

Mean concentrations (nmol/L), standard deviations (SD, nmol/L), and precision (% CV) are shown for seven serum samples analyzed in replicates of five (intra‐assay) or nine (inter‐assay).

**Table 2:** Characterization of assay specificity

Sample matrix	Sample ID	w/o competition c (nmol/L)	With competition c (nmol/L)	Recovery (%)
Serum	s1	1.5	0.2	84
Serum	s2	2.0	0.0	100
Serum	s3	2.6	0.0	100
EDTA plasma	e1	1.4	0.0	100
EDTA plasma	e2	1.8	0.0	100
EDTA plasma	e3	2.1	0.0	100
Citrate plasma	c1	1.9	0.0	100
Heparin plasma	h1	2.2	0.0	100
			Mean recovery	98

Note

sNRP1 concentrations (nmol/L) in serum and plasma samples were determined with or without (w/o) prior addition of a surplus of coating antibody acting as competitor. Recovery (%) was assessed, and mean recovery for all samples is shown.

The assay parameters met the international standards of acceptance according to ICH guidelines.

### 3.3 Mapping of linear antibody epitopes and discussion of assay cross‐reactivities

**Figure 6:**
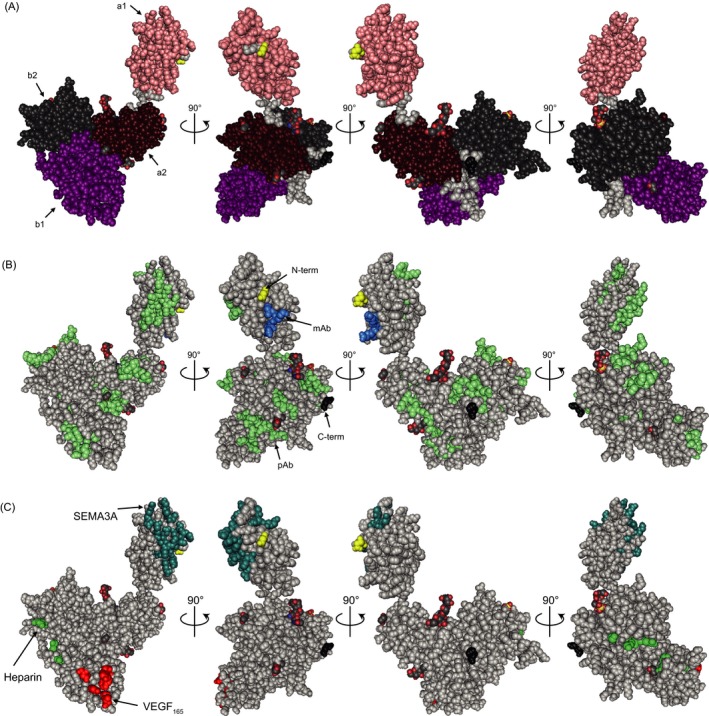
Surface representation of mouse NRP1 with stepwise rotation of 90°. A, extracellular domains of NRP1 (a1a2b1b2) are shown (N‐terminus of a1 domain: yellow, C‐terminus of b2 domain: black). B, Linear epitopes of the polyclonal coating antibody (green) and the monoclonal detection antibody (blue) were mapped with microarray technology and are depicted. C, Binding regions of the sNRP1 ligands SEMA3A (turquois), VEGF‐A_165_ (red), and heparin (green) are indicated

The full extracellular domain of human NRP1 is not resolved so far; therefore, a three‐dimensional X‐ray model of mouse NRP1^17^ was used to show the extracellular domains of NRP1 (a1a2b1b2; Figure 6A). The model was further taken to display the linear epitopes of antibodies employed in the human sNRP1 assay (Table 3; Figure 6B). In detail, the polyclonal coating antibody binds to 17 major linear epitopes (green) that are distributed over all extracellular domains of sNRP1. Three epitopes are in the a1 domain, three in the a2, four in the b1, and five in the b2 domain (Table 3; Figure 6B). Two more epitopes are located between b2 and MAM domains (data not shown). The linear epitope of the monoclonal detection antibody is shown in blue and is situated in the a1 domain adjacent to the N‐terminus (Table 3; Figure 6B).

**Table 3:** Linear epitopes of ELISA assay antibodies

Antibody function	Clonality	Epitope ID	Epitope sequence
coating	polyclonal	p_e1	NFNPHFDLE
coating	polyclonal	p_e2	KYDYVEVF
coating	polyclonal	p_e3	KIAPPPVV
coating	polyclonal	p_e4	SGVIK
coating	polyclonal	p_e5	SNPPGGMF
coating	polyclonal	p_e6	GRIRSSSGILSMVFYTD
coating	polyclonal	p_e7	EALGM
coating	polyclonal	p_e8	GEIHSDQITA
coating	polyclonal	p_e9	RLNY
coating	polyclonal	p_e10	KPATWETGIS
coating	polyclonal	p_e11	VSGLI
coating	polyclonal	p_e12	SSNQGDR
coating	polyclonal	p_e13	PPAPHSY
coating	polyclonal	p_e14	IDLGEEKI
coating	polyclonal	p_e15	PELRTF
coating	polyclonal	p_e16	GTTVLATE
coating	polyclonal	p_e17	VIDSTIQSGI
detection	monoclonal	m_e1	IKIE

Note

Sequences of linear epitopes of the polyclonal coating antibody and the monoclonal detection antibody employed in the sNRP1 ELISA were mapped by microarray technology and are shown.

The homology of NRP1 to other human proteins is low. According to BLAST analysis, highest sequence identity of 53% was found for NRP2 isoform 6. The non‐neuropilin hit with the highest identity of 40% was found for neuropilin and tolloid‐like protein 1 (NETO1). However, the NRP1 epitope of the monoclonal antibody (IKIE; Table 3) is not conserved on NRP2 and NETO1 (data not shown); therefore, assay cross‐reactivity with these molecules can be excluded. In contrast, analysis of the homology between human, mouse, and rat NRP1 revealed high sequence identities of >90% between the three species, with a high conservation of the monoclonal antibody epitope. Still, mouse and rat samples did not react in the novel sandwich ELISA (data not shown).

### 3.4 SEMA3A or VEGF‐A_165_ binding interferes with NRP1 quantification

**Figure 7:**
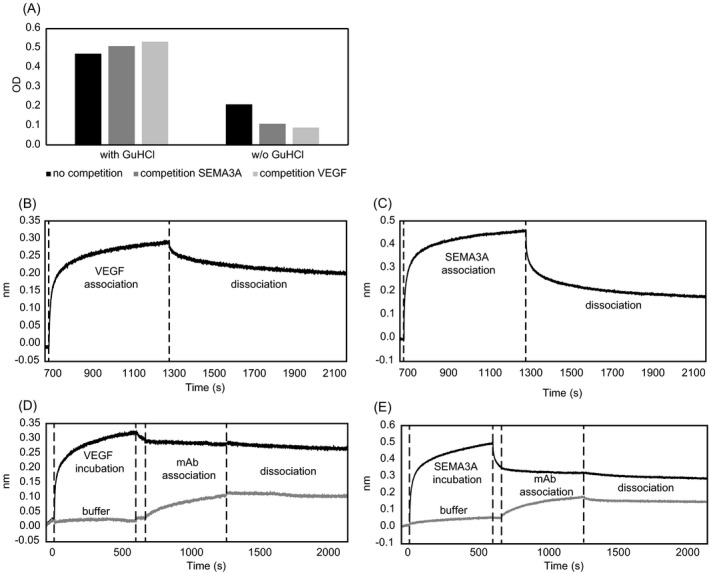
Analysis of sNRP1 interferants in ELISA (A) and bio‐layer interferometry (B‐E). A, Recombinant sNRP1 with or without (w/o) GuHCl pre‐treatment was tested by competition with SEMA3A or VEGF‐A_165_ (*y*‐axis: OD values). B‐E, Sensor‐bound sNRP1 was incubated with VEGF‐A_165_ or SEMA3A to analyze dissociation rates (B, C) or to test for binding influence on the monoclonal ELISA detection antibody (D, E). Signal intensities are shown on the *y*‐axes (nm) vs the time (s, *x*‐axes)

To test if the NRP1 ligands SEMA3A or VEGF‐A_165_, that bind near the linear antibody epitopes (Figure 6C), have influences on sNRP1 quantification in the assay, competition experiments with the two ligands were performed. Therefore, recombinant sNRP1 spiked in sNRP1‐depleted serum matrix was pre‐treated with GuHCl according to the assay protocol and diluted 1:10 with assay buffer. The prior addition of SEMA3A or VEGF‐A_165_ showed no competition effect with any of the tested competitors (CV of the three measurements: 6%; Figure 7A). However, when the assay was performed aside of the protocol with exclusion of the GuHCl pre‐treatment step two effects were observed. First, the obtained signal was suppressed by matrix interferences, and second, an additional inhibiting effect could be observed for both ligands, SEMA3A or VEGF‐A_165_ (CV of the three measurements: 47%; Figure 7A).

To further investigate sNRP1 binding behavior of the two ligands and their influence on monoclonal detection antibody binding to sNRP1 in the absence of GuHCl pre‐treatment and in a protein‐free environment, bio‐layer interferometry measurements were performed. Sensor‐bound sNRP1 was incubated with VEGF‐A_165_ or SEMA3A, revealing slow dissociation of VEGF‐A_165 _from sNRP1 (Figure 7B). SEMA3A‐sNRP1 binding was less stable, with an initial fast dissociation rate that stabilized after five minutes, but still SEMA3A dissociated much faster than VEGF‐A_165_ (Figure 7C). Next, sNRP1 binding of the monoclonal detection antibody was analyzed with and without prior ligand incubation. Both ligands heavily influenced binding of the monoclonal antibody (Figure 7D, 7). Thereby, high‐affinity VEGF‐A_165_ binding to sNRP1 almost completely prohibited antibody binding (Figure 7D). SEMA3A, which binds to sNRP1 in close vicinity of the monoclonal antibody epitope, also inhibited antibody binding (Figure 7E).

## 4 DISCUSSION

Soluble neuropilin‐1 is described as a highly interactive molecule, with 48 unique physical interactors known so far.^18^ High‐affinity bound ligands may therefore interfere with quantification systems.

In the current study, we describe the development and performance of a highly specific, accurate, and robust human sNRP1 sandwich ELISA that uses a polyclonal sheep anti‐human NRP1 capturing antibody and a labeled monoclonal mouse anti‐human NRP1 detection antibody. Linear epitopes of the employed antibodies were mapped, and sequences are shown to enable a better insight into the antibody‐antigen reaction. The assay has a calibrator range between 0.375 nmol/L and 12 nmol/L, and a quantification limit of 0.09 nmol/L. Assay parameters met the international standards of acceptance according to ICH guidelines.

Interestingly, during ELISA development we found out that our assay setup was indeed affected by an unknown interferant. This interferant was shown to partially overlap with antibody binding sites as low sNRP1 concentrations could still be measured. In fact, it was the epitope of the monoclonal detection antibody that was affected as longer incubation of the detection antibody with the sample displaced the factor and released sNRP1. To simulate this disturbing factor, we used the NRP1 ligand with strongest reported affinity, VEGF‐A_165_ (*K*_D_ = 3 nM),^14^ and showed in sensor‐based experiments that incubation of NRP1‐bound VEGF‐A_165_ with a high concentration of the strong chaotrope GuHCl completely removed VEGF‐A_165_ from sNRP1. To enable interference‐free quantification of sNRP1 in our ELISA, we included this finding and developed a sample pre‐treatment method based on 3 mol/L GuHCl.

Although multiple NRP1 ligands are described, binding sites on NRP1 are only known for few ligands. The first NRP1 ligand that was identified was SEMA3A,^12^, ^19^ which interacts with NRP1 at two sites, with a specificity‐determining contact in the a1a2 domain and an affinity‐relevant contact in the b1 domain of NRP1.^13^, ^20^ The SEMA3A binding site in the a1 domain of NRP1 was reconstructed by combining results from the described epitope of a SEMA3A‐blocking NRP1‐specific Fab described by Appleton et al^13^ and from site‐directed mutagenesis studies by Gu et al.^3^ The thereby resulting binding site lies in close vicinity of the linear epitope of the monoclonal detection antibody, which makes SEMA3A a potential interferant that may hinder the monoclonal detection antibody from NRP1 binding due to a binding site overlap or due to steric hindrance when the novel assay is performed without GuHCl pre‐treatment. Another main ligand of sNRP1 is the heparin‐binding VEGF isoform VEGF‐A_165_, which has a higher affinity to NRP1 than SEMA3A.^21^ The binding site in exon 7 of VEGF‐A_165_ was resolved by crystallization.^14^, ^15^ It lies opposite of the SEMA3A binding site, that is, in the b2 domain, next to an epitope of the polyclonal antibody.^4^ VEGF‐A binding to NRP1 is further enhanced by heparin, whose binding residues were identified in the b1b2 region of NRP1 by mutational studies^15^ and do not overlap with resolved linear antibody epitopes. Due to the steric separation of the b region binding ligands and the monoclonal antibody epitope, it seems quite unlikely that VEGF‐A or heparin are the disturbing interactors. However, heparin further promotes dimerization of NRP1 or of the NRP1‐VEGF complex. Hence, a created complex would not directly affect the a1 domain of NRP1 carrying the monoclonal antibody epitope, but monoclonal antibody binding may sterically be hindered.

We could show that high‐affinity VEGF‐A_165_ binding to sNRP1 almost completely prohibited monoclonal antibody binding, which indicates for steric hindrance of the monoclonal antibody due to sNRP1‐VEGF‐A_165_ complex formation. Consequently, the majority of VEGF‐A_165_‐bound sNRP1 would not be detected in the sandwich ELISA without GuHCl pre‐treatment. SEMA3A, which binds to sNRP1 in close vicinity of the monoclonal antibody epitope, also inhibited antibody binding. However, the fast dissociation of SEMA3A from sNRP1 may allow the monoclonal antibody to bind and further displace SEMA3A from sNRP1, which may account for the time‐related sNRP1 increase in endogenous samples without GuHCl pre‐treatment.

In conclusion, we could demonstrate that it is possible to release sNRP1 from a distracting factor in the sample matrix by treating the samples with the chaotrope GuHCl. The implication of this procedure into the ELISA protocol of the described sandwich ELISA now allows quantification of total sNRP1, that is, free as well as separated sNRP1 that was ligand‐bound before. Total sNRP1 measurement discriminates our assay from the R&D Systems Quantikine Human Neuropilin‐1 ELISA. The R&D assay is described by the manufacturer to be unaffected by interferences as tested molecules like NRP2, SEMA3A, SEMA3E, and Plexin A4 did not interfere with the assay performance. However, this minimal selection of competitors did not even include the main NRP1 ligand VEGF‐A, which makes a general exclusion of interferences impossible.

Beyond this present example, interferences are a frequent problem in the quantification of ligands that circulate with concentrations in a low molar range. The described method may therefore help to overcome such obstacles also in the development of other immunoassays.
